# β3 integrin expression is required for invadopodia-mediated ECM degradation in lung carcinoma cells

**DOI:** 10.1371/journal.pone.0181579

**Published:** 2017-08-02

**Authors:** Rafael Peláez, Xabier Morales, Elizabeth Salvo, Saray Garasa, Carlos Ortiz de Solórzano, Alfredo Martínez, Ignacio M. Larrayoz, Ana Rouzaut

**Affiliations:** 1 Department of Oncology, Center for Applied Medical Research CIMA, Pamplona, Spain; 2 Biomarkers and Molecular Signaling Group, Neurodegenerative Diseases Area, Center for Biomedical Research of La Rioja, CIBIR, Logroño, Spain; 3 Laboratory of Preclinical Models and Analytical Tools, Division of Solid Tumors and Biomarkers, Center for Applied Medical Research and CIBERONC, Pamplona, Navarra, Spain; 4 Department of Immunology and Immunotherapy, CIMA, Pamplona, Navarra, Spain; 5 Oncology Area, Center for Biomedical Research of La Rioja, CIBIR, Logroño, Spain; 6 Department of Biochemistry and Genetics, University of Navarra, Pamplona, Spain; Seoul National University College of Pharmacy, REPUBLIC OF KOREA

## Abstract

Cancer related deaths are primarily due to tumor metastasis. To facilitate their dissemination to distant sites, cancer cells develop invadopodia, actin-rich protrusions capable of degrading the surrounding extracellular matrix (ECM). We aimed to determine whether β3 integrin participates in invadopodia formed by lung carcinoma cells, based on our previous findings of specific TGF-β induction of β3 integrin dependent metastasis in animal models of lung carcinoma. In this study, we demonstrate that lung carcinoma cells form invadopodia in response to TGF-β exposure. Invadopodia formation and degradation activity is dependent on β3 integrin expression since β3 integrin deficient cells are not able to degrade gelatin-coated surfaces. Even more, transient over-expression of SRC did not restore invadopodia formation in β3 integrin deficient cells. Finally, we observed that blockade of PLC-dependent signaling leads to more intense labeling for β3 integrin in invadopodia. Our results suggest that β3 integrin function, and location, in lung cancer cells are essential for invadopodia formation, and this integrin regulates the activation of different signal pathways necessary for the invasive structure. β3 integrin has been associated with poor prognosis and increased metastasis in several carcinoma types, including lung cancer. Our findings provide new evidence to support the use of targeted therapies against this integrin to combat the onset of metastases.

## Introduction

Metastasis is the main cause of cancer-related death [1]. For metastasis to occur, cancer cells must leave their primary niche and move towards target organs. This journey requires overcoming tissue barriers otherwise intended to constrain cells from escaping their physiological niche. In these processes, cancer cells need to acquire matrix degrading phenotypes in which the development of stiff membrane-derived structures and the activation of matrix metalloproteinases play prominent roles as tools to drill new paths.

Recently, actin-driven matrix degradative structures called invadopodia have been described in cancer cells. These structures present strong similarities in composition and function with the podosomes described in non-transformed cells, such as osteoclasts and macrophages. Both invadopodia and podosomes are characterized by the presence of a core of actin bundles perpendicularly oriented to the substrate and surrounded by adhesion molecules such as integrins. Besides, they are endowed with strong degradative activity exerted by matrix metalloproteinases such as Mt1-MMP, Mmp2, and Mmp9 [2]. There are some differences between podosomes and invadopodia; podosomes are small, abundant and dynamic, while invadopodia constitute less dynamic structures that are usually present in lower number [3]. Invadopodia formation in primary human tumors or in cancer metastasis is not completely accepted yet. However, both *in vitro* and *in vivo* mouse models of cancer provide strong evidences that support their existence, and suggest targeting their formation as a new approach to prevent tumor spread [4].

Several soluble molecules such as EGF, HGF, and TGF-β have been associated with invadopodia formation. Additionally, mechanical constrains derived from the underlying extracellular matrix (ECM), such as rigidity, density, and dimensionality are also important in the development of these structures. Thus, invadopodia assembly requires the integration of signals from the ECM, sensed by integrins on the cell surface, with cytoskeletal rearrangements which determine invadopodia number, size, proteolytic activity, and half-life [3,5,6].

Integrins are a family of receptors with high functional redundancy, composed by 24 different αβ heterodimers, arranged in combinations of 18 different α-subunits and 8 β-subunits [7]. Integrin activation by their binding to extracellular ligands stimulates a switch from low to high affinity conformation [8], and triggers signaling cascades that lead to the reorganization of the cytoskeleton [9,10]. Although several studies have demonstrated the relevant role of β1 and β3 integrins in the formation of the podosome adhesive ring, their presence in tumor invadopodia is still controversial [11]. In this sense, some reports had previously demonstrated the presence of β1 and α5 integrin surrounding the invadopodia core in breast cancer cells [12], and of α6β1 and α3β1 integrins in invadopodia formed *in vitro* by melanoma cells [13]. Additionally, the presence of αvβ5 integrin has been described in invadopodia formed *in vitro* by oral squamous cell carcinoma cells [14].

In a previous work, we established a relationship between increments in β3 integrin expression induced by TGF-β exposure and metastasis in mouse models of lung carcinoma [15], and the switch in the cell migration phenotype after integrin blockade [16]. Although β3 integrin has been described as a non-essential component of osteoclast and epithelial cell podosomes [17,18,19], some authors propose that this integrin is relevant for bone mass increments [20,21]. The role of β3 integrin in the development and activity of invadopodia in transformed cells is even more controversial and remains unclear [22,23].

In this work, we aim to evaluate the role that β3 integrin exerts in invadopodia formation by non small cell lung cancer (NSCLC). We demonstrate that lung carcinoma cells form invadopodia in response to TGF-β, and that this formation depends on β3 integrin expression. Our results suggest a transitory, but vital, function for β3 integrin in invadopodia formation in lung carcinoma cells.

## Material and methods

### Cell culture

The human NSCLC cell lines H157, H1299, and A549 were purchased from the American Type Culture Collection (ATCC, LGC-Promochem SL, Barcelona, Spain). Cells were grown at 37°C, 5% CO_2,_ in RPMI medium (LONZA, Barcelona, Spain) supplemented with 10% FetalClone III (HyClone) from Thermo Scientific (Waltham Massachusetts, USA) and 100 units/ml penicillin-streptomycin. Cell lines were authenticated by PCR amplification of genomic DNA using primers for the specific CDKN2A mutation (c.205 G>T, in exon 2) and a KRAS mutation (c.34 G>C, in exon 2), followed by sequencing of the PCR products.

### Antibodies and reagents

Recombinant Human TGF-β was purchased from R&D (R&D Systems, Minneapolis, USA). TGF-βRI chemical inhibitor SB431542, Geneticin (G418), Puromycin dihydrochloride antibiotic, and Hygromycin B were obtained from Sigma-Aldrich (Steinheim, Germany), MMP inhibitor GM6001 was from Millipore (Billerica, MA, USA).

Antibodies: Fak (rabbit polyclonal antibody; #3285), pTyr397-Fak (rabbit polyclonal antibody; #3283), Src (mouse monoclonal antibody; #2110), pTyr416-Src (rabbit polyclonal antibody; #2101) and Smad2/3 (rabbit polyclonal antibody; #3102) were purchased from Cell Signaling (Danvers, MA, USA). Anti-β-actin (mouse monoclonal antibody; A5441) and pTyr421-Cortactin (rabbit polyclonal antibody; SAB4504372-100UG) were from Sigma-Aldrich. Phosho-Smad2 (rabbit polyclonal antibody; ab3849), anti-cortactin 4F11 (mouse monoclonal antibody; 05–180) and Integrin β3 (mouse monoclonal antibody; MAB 2023-z) antibodies were from Millipore. Mmp2 (rabbit polyclonal antibody; RB-1537-P0) and Mmp9 (rabbit polyclonal antibody; RB-1539-P0) were from Neomarkers/Thermo Scientific. Tks5 ([FishM-300] rabbit polyclonal antibody; sc-30122), anti-mouse IgG-HRP (goat polyclonal antibody; sc-2005) and anti-rabbit IgG-HRP (goat polyclonal antibody; sc-2004) were from Santa Cruz Biotechnology (Dallas, Texas, USA). Mmp14 ([Mt1-MMP] rabbit monoclonal antibody; ab51074) and Vinculin (mouse monoclonal antibody; ab18058) were from Abcam, (Cambridge, UK). Immunofluorescence secondary antibodies Alexa Fluor-594 donkey anti-rabbit (A-21207), Alexa Fluor-546 goat anti mouse (A-11003), Alexa Fluor-594 donkey anti-mouse (A-21203), Alexa Fluor-647 donkey anti-mouse (A-31571) and Alexa Fluor-647 donkey anti-rabbit (A-31573) were from Life Technologies (Invitrogen, Barcelona, Spain). Isotype control IgG1 ([Mg1-45] 401202) was from Biolegend (London, UK)

### Cell treatments

H157 NSCLC cells were cultured in serum-free RPMI with 2 ng/ml human recombinant TGF-β for 2 or 5 days. The medium was replaced and fresh cytokine was added every 48 hours. For TGF-β blocking experiments, tumor cells were incubated with 10 mM SB431542 30 minutes before TGF-β treatment. MMPs were inhibited by incubation during cell adhesion with 25 μM GM6001. For PLC inhibition, and subsequently PIP_2_ increase, cells were incubated in 10 mM neomycin trisulphate 30 minutes before seeding.

### Tumor cell transfection

H157 cells (3×10^5^ cells/ml) were transfected with 1 μg of a scrambled RNA or a HuShTMshRNA Plasmid Panels-29mer targeting β3 integrin (Origene, Rockville, MD, USA) in Opti-MEM medium (Invitrogen) using a Bio-Rad Gene Pulsar I electroporator (Bio-Rad, Berkeley, CA, USA). Stable β3 integrin-silenced clones or cells expressing a non-specific scrambled RNA sequence were selected by culturing cells in the presence of 1.5 μg/ml puromycin dihydrochloride.

For β3 integrin re-expression, 3×10^5^ H157-shRNA β3 integrin cells were transfected with 1 μg of the pcDNA3-Higro+ or pcDNA3-Higro+-hβ3 integrin plasmid [24] using FuGENE 9 Transfection Reagent (Roche, Molecular Biochemicals, [Mannheim, Germany]), following manufacturer’s instructions. β3 integrin re-expression and mock cells were selected by culturing cells in the presence of 500 μg/ml hygromicin B.

To generate SRC-GFP transient expression in H157, and H157shRNA β3 integrin, cell lines, 3×10^5^ cells were transfected with 1 μg of the pEGFP-SRC plasmid [25] and pEGFP-c1 using FuGENE 9 Transfection Reagent as above. Clones were selected by culturing cells in the presence of 1mg/ml G418.

### Flow cytometry

β3 Integrin detection was made as previously described [26] by flow cytometry using a FACScan (Becton-Dickinson System). Data were processed using CellQuest software (BD-Bioscience, Madrid, Spain).

### Western blot

Total cell protein extracts were prepared using RIPA buffer as previously described [26]. All protein samples were electrophoresed in a SDS-polyacrylamide gel and transferred onto PVDF membranes. PVDF membranes were blocked for 2 hours with 5% non-fat milk or 5% BSA in TBS containing 0.1% Tween-20, and then incubated overnight at 4°C with primary antibodies. Membranes were washed three times with TBS-Tween 0.05% and incubated with HRP-conjugated anti-rabbit IgG or anti-mouse IgG secondary antibodies for 1 hour at room temperature in 5% non-fat dry milk in TBS-Tween. Immunoreactive bands were developed with Lumi-light^PLUS^ Western Blotting Kit (Roche, Molecular Biochemicals) and visualized using X-ray films (Amersham Hyperfilm ECL, GE Healthcare, Buckinghamshire, UK).

### Adhesion assays

5x10^4^ cells were seeded onto 8 well Nunc^TM^ Lab-Tek^TM^ chambered coverglasses covered with gelatin from 1 to 120 hours at 37°C (Life Technologies, Invitrogen), and fixed with 4% PFA for 10 minutes. Cell adhesion and morphology was observed by immunofluorescence detection of vinculin with a confocal microscopy, as we describe later. We considered non adhered cells those that are not completely fully spread and with >7μm height measured by Z-Stacks reconstructions

### 2D invadopodia gelatin degradation assay

Nunc^TM^ Lab-Tek^TM^ chambered coverglasses (Thermo Scientific) were coated with 50 μg/ml poly-L-lysine for 15 minutes, washed with PBS, and cross-linked with 0.5% glutaraldehyde for 15 minutes. Glutaraldehyde was removed and a 100-μl drop of 1 mg/ml Alexa Fluor 488-conjugated gelatin (Life Technologies, Invitrogen, Barcelona, Spain) was added for 10 minutes to each coverglass. The fluorescent signal was quenched with 5 mg/ml sodium borohydride for 3 minutes followed by a PBS wash. Finally, coverglasses were sterilized with 70% ethanol for 5 minutes and covered with cell growth medium 1 hour before use. To detect invadopodia formation, 100 cells/μl cells were seeded over cross-linked fluorescent conjugated matrix for 1 to 120 hours. The percentage of cells showing degradation was quantified by microscopy inspection. A cell was considered positive for the presence of invadopodia when it has at least one actin/cortactin enriched cell protrusion inside of an individual or sets of degraded areas in the ventral side of the cells.

### 3D invadopodia matrix degradation assay

To visualize invadopodia matrix degradation, 5x10^4^ cells were embedded in 200 μl of Matrigel containing 25 μg/ml DQ-type I collagen (Life Technologies, Invitrogen) and mixed gently before plating in 8 wells Nunc^TM^ Lab-Tek^TM^ chambered coverglasses (Thermo Scientific) and incubating at 37°C for 2 hours. Then, medium supplemented with 10% FetalClone III and 2 ng/ml TGF-β was added on top of the Matrigel. After 72 hours, cells were fixed with 4% paraformaldehyde (PFA), permeabilized with 0.5% saponin, blocked with 5% bovine serum albumin (BSA), and F-actin was stained with TRICT-conjugated phalloidin (Sigma-Aldrich). Invadopodia were identified as actin-rich protrusions that colocalized with matrix degradations (enhanced DQ-type I collagen signal). 3D reconstructions were generated from Z-stacks using Volocity Software (Perkin Elmer, Waltham, MA).

### Confocal microscopy imaging

Cells were seeded onto 8 well Nunc^TM^ Lab-Tek^TM^ chambered coverglasses covered with gelatin from 1 to 120 hours at 37°C (Life Technologies, Invitrogen), and fixed with 4% PFA for 10 minutes. Afterwards, cells were permeabilized with 0.5% Triton X-100 in PBS for 5 minutes. Non-specific binding was blocked by incubation with 3% BSA (Sigma-Aldrich) for 30 minutes at room temperature. Incubation with a specific primary antibody was carried out overnight with Mmp14, Tks5, β3 integrin or vinculin. Anti-cortactin 4F11 required an incubation of 48 hours. Samples were rinsed with PBS and subsequently incubated for 1 hour at room temperature with Alexa secondary antibodies. F-actin was stained with TRITC-conjugated phalloidin or phalloidin CruzFluor^TM^ 647 conjugate (Santa Cruz Biotechnology). For image acquisition, LSM 510 META (Zeiss, Jena, Germany) inverted confocal microscope equipped with a 40xP-Neofluar (NA 1.3) and 63x Plan-Apochromat objective (NA1.4) were used. Images were acquired using the Zeiss software (Aim4). Three dimensional reconstructions were made using Volocity Software (Perkin Elmer) and Imaris Scientific 3D/4D Image Processing analysis software (Bitplane AG, Zurich, Switzerland). Images analyses were performed using ImageJ software and LSM image analyzer software from Zeiss. Invadopodia degraded areas were measured with ImageJ software by a manual threshold cut off of non fluorescent areas to allow the binary quantification.

In integrin blockade assay, H157 cells were blocked using anti-αvβ3 integrin (Millipore) and IgG1 isotype control antibodies at 10 μg/ml for 1 hour before cell seeding on Oregon Green 488-conjugated gelatin.

### Statistical analysis

Normal distribution of data was analyzed by Kolmogorov-Smirnov test. Results were compared by the Student’s t-test or one-way ANOVA followed by post-hoc analyses. Non-parametric data was analyzed using Kruskal-Wallis and Mann-Whitney U tests. In all cases, test results were considered significant when the probability was smaller than 0.05 (p <0.05) *, very significant (p <0.01) **, and highly significant when the probability was smaller than 0.001 (p <0.001) ***. All analyses were performed using SPSS 15.0 and graphic representations were made with Graph Pad Prism 5 software.

## Results

### β3 integrin expression is necessary to induce gelatinolytic activity in NSCLC cells

In our previous work, we found that β3 integrin was one of the ECM receptors highly expressed in NSCLC cells after exposure to TGF-α. Importantly, silencing of this integrin dramatically reduced the incidence of lymph node metastasis in an orthotopic model of lung carcinoma developed in mice [15].

**Fig 1:**
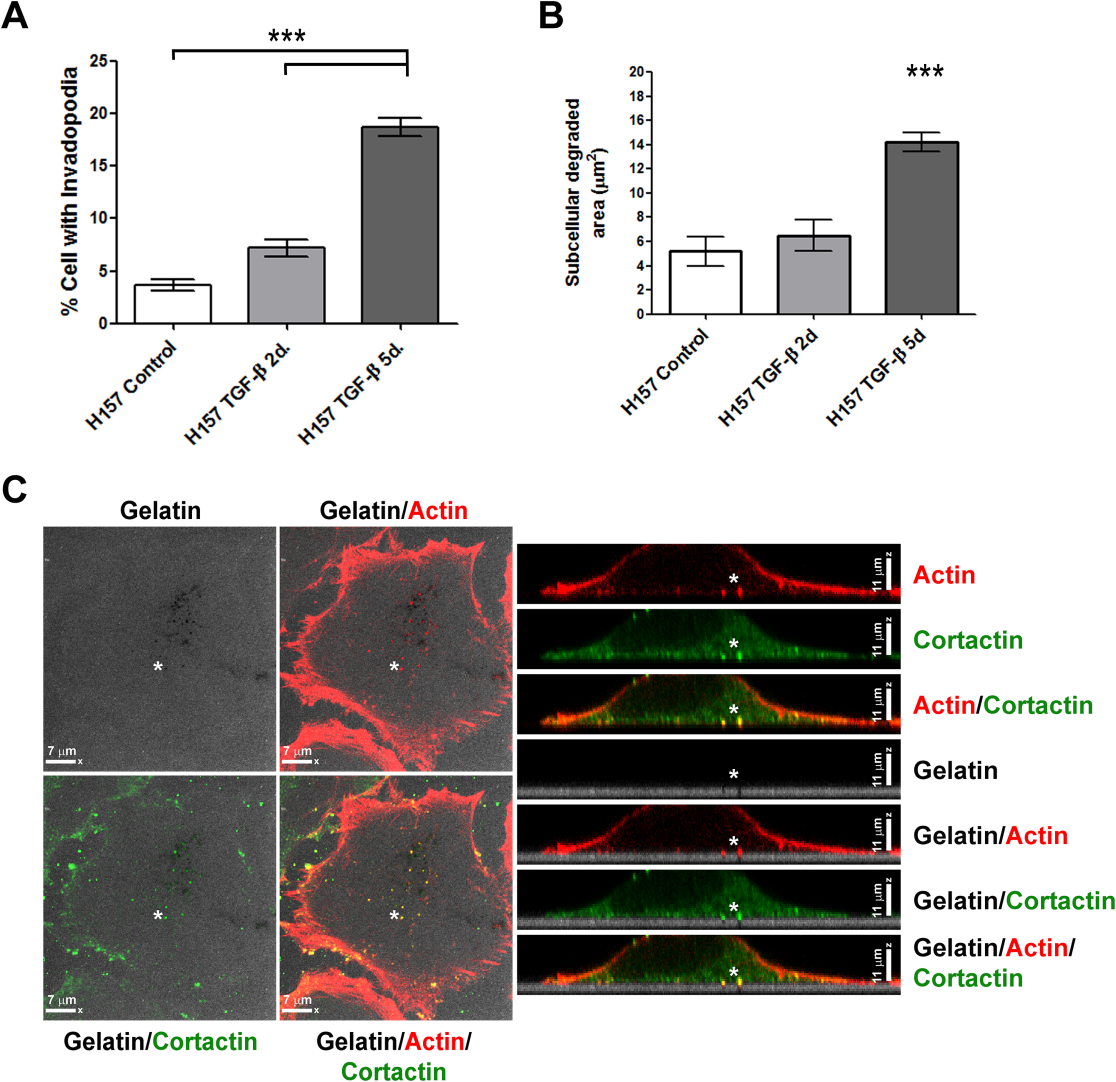
H157 lung squamous carcinoma cells present incremented invadopodia activity after TGF-β treatment. **(A)** Percentage of H157 NSCLC cells with gelatin-degrading invadopodia. Cells had been exposed for 2 and 5 days to TGF-β were seeded onto green fluorescent-labelled gelatin for 20 hours. Data represent mean ± SEM of cells with active invadopodia obtained from at least three different experiments. Three fields and a total of one hundred cells were analysed per experiment. Significant differences were calculated by one-way ANOVA followed by post-hoc Tukey´s tests for multiple comparisons. *** p<0.001. **(B)** Quantification of total degraded area under cells with active invadopodia. Cells were exposed for 2 and 5 days to TGF-β and then seeded onto green fluorescent-labelled gelatin for 20 hours. Data represent mean ± SEM of total degraded areas in μm^2^. For statistical analysis one-way ANOVA followed by post-hoc Tukey´s tests for multiple comparisons was used. *** p <0.001. **(C)** Microphotographs from representative Z-stacks obtained by confocal-laser microscopy are shown. Cells were cultured on 488-gelatin films (grey). Actin (red) and cortactin (green) were detected by immunofluorescence, with donkey anti-mouse alexa 647 and Phalloidin-TRICT, in invadopodia formed on gelatin-degraded areas. Scale bars 7 μm in XY-image and 11 μm in Z-image. White asterisks point degradation sites.

Now, we performed experiments in order to study whether β3 integrin participates in the development of invadopodia produced by H157 NSCLC cells. For this aim, we first established an *in vitro* model, in which invadopodia formation was incremented over baseline by treating cells with TGF-β (20 ng/ml) for 5 consecutive days. This cytokine has been described to induce invadopodia formation in cancer cells [27]. Fig 1A and S1A Fig show how the formation of gelatin-degrading and actin-enriched structures was significantly higher in H157 NSCLC cells after 5 days of TGF-β treatment. Moreover, this treatment also incremented the extension of total degraded areas (Fig 1B). These structures were identified as invadopodia since they presented cortactin and actin cores, and showed gelatin degradation underneath actin puncta (Fig 1C). Pretreatment of cells with the specific TGF-βRI kinase inhibitor, SB431542, returned matrix degradation to baseline (S1B Fig).

In addition, the inhibition of metalloproteinase activity by treatment with GM6001, prevented gelatin degradation supporting the presence of metalloproteinases in these structures (S1C and S1D Fig). These results were reinforced by the detection of active forms of Mmp2 by Western blotting in cell extracts obtained from TGF-β exposed cells (S1E Fig).

**Fig 2:**
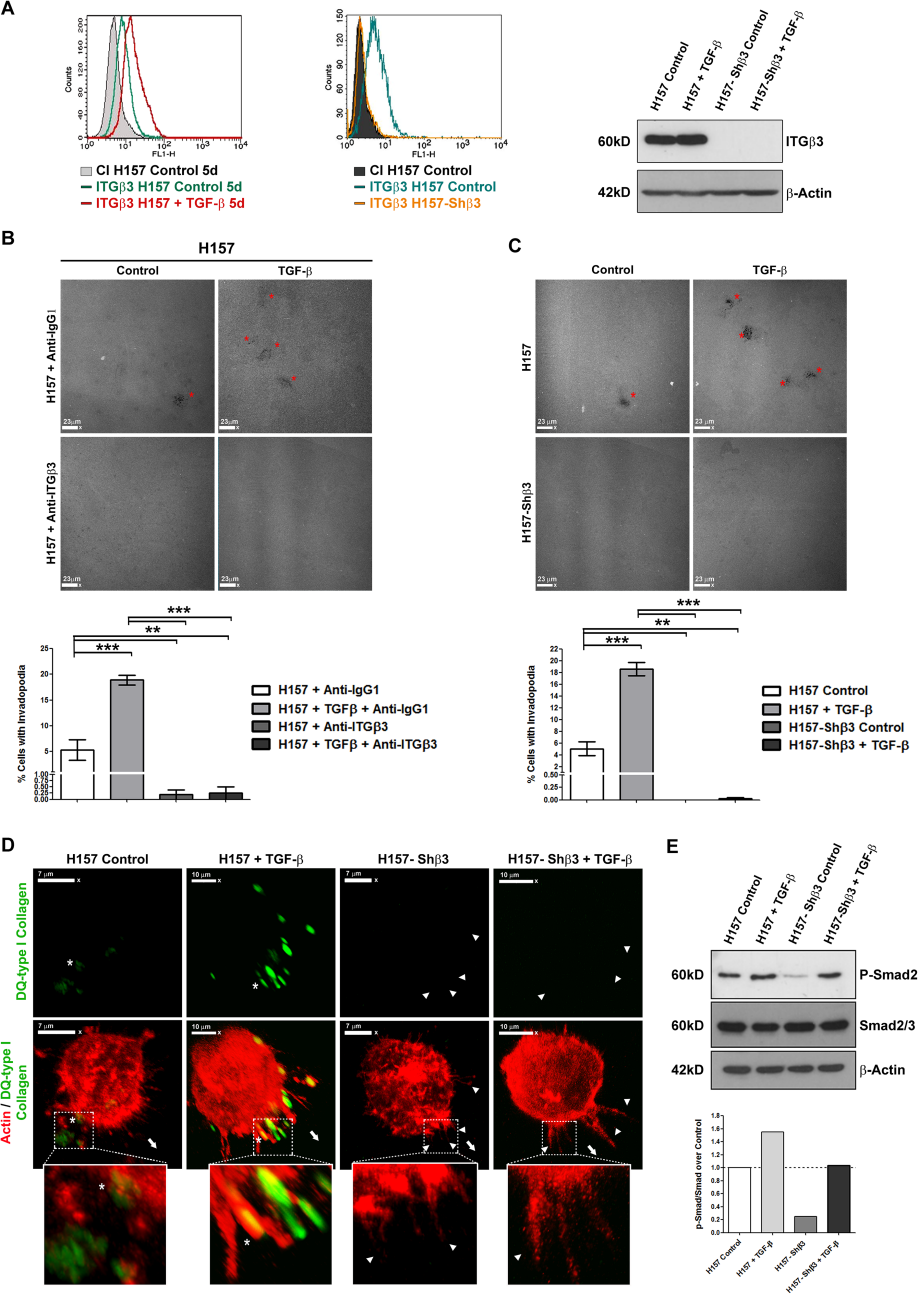
β3 integrin blockade or silencing inhibits invadopodia formation by H157 NSCLC cells. **(A)** Flow cytometry and Western blot analysis of β3 integrin expression in control and TGF-β treated H157 cells before and after β3 integrin silencing with a specific shRNA. β-Actin was used as Western blot loading control. **(B)** Quantification of the percentage of H157 NSCLC cells with gelatin-degrading invadopodia after β3 integrin blockage by preincubation with anti-β3 integrin mAbs (1 μg of antibody for 2 hours before the experiment). Cells were cultured as in Fig 1. Isotypic non-specific IgG treatment was included as control. Data represent mean ± SEM of four different experiments in which at least three fields and a total of 100 cells per experiment were analysed. For statistical analyses a one-way ANOVA followed by post-hoc Tukey´s tests for multiple comparisons was used. ** p< 0.01 and *** p< 0.001. Panels show representative microphotographs of gelatin-degraded areas obtained in each experimental condition. Scale bars 23 μm. Red asterisks indicate degradation sites. **(C)** Quantification of the percentage of cells with gelatin-degrading invadopodia in H157 cells expressing or not β3 integrin and treated or not with TGF-β. Cells were cultured as in Fig 1. Data represent mean ± SEM of four different experiments in which at least three fields and a total of 100 cells per experiment were analysed. For statistical analysis one-way ANOVA followed by post-hoc Tukey´s tests for multiple comparisons was used. ** p< 0.01, *** p< 0.001. Panels show representative microphotographs of gelatin-degraded areas obtained in each experimental condition. Scale bars 23 μm. Red asterisks indicate degradation sites. **(D)** 3D reconstructions obtained from confocal-laser microscopy images captured from H157 cells grown in DQ-collagen matrices for 72 hours. Actin (red) and DQ-collagen degradation areas (green) are shown in wild type and β3 integrin-silenced cells treated or not with TGF-β for 5 days. Scale bar 10 μm and 7μm, as indicated. White asterisks indicate examples of collagen degradation sites associated with actin protrusion tips, whereas white arrowheads point to actin puncta without associated degradation. Big white arrow signals cell orientation. The white boxes show a magnified image of one selected. **(E)** Western blot analysis and signal quantification by densitometry of total and phosphorylated Smad2/3 levels in H157 cells and β3 integrin silenced cells treated, or not, with TGF-β. β-Actin was used as loading control.

Next, we tested whether the expression of β3 integrin was necessary for invadopodia formation in H157 NSCLC cells. To this end, we performed gelatin-degrading assays using β3 integrin silenced cells. Functional blocking of β3 integrin was accomplished by gene silencing with a shRNA specific plasmid (Fig 2A and 2C) or with specific β3 integrin mAbs (Fig 2B). Results showed that β3 integrin functional blocking (Fig 2B) or genetic silencing (Fig 2C), completely abolished the formation of invadopodia in H157 cells. To ascertain whether these structures were also formed in restrictive 3D environments, we performed matrix-degrading assays using cells embedded in DQ-collagen matrices. Fig 2D shows the formation of tridimensional actin enriched structures associated with fluorescent degraded DQ collagen in control and TGF-β treated cells expressing or not β3 integrin.

Interestingly, β3 integrin silenced cells protrude 3D actin enriched structures in the 3D matrix but they were unable to degrade DQ collagen, pointing at the need of this integrin for invadopodia maturation and proteolytic activity. We wanted to ascertain whether β3 integrin silencing will abrogate signaling through TGF-β pathway. In fact, it has been demonstrated the existence of a feedback loop in which β3 integrin participates in TGF-β activation [28]. As shown in Fig 2E, β3 integrin deficient cells present lower phosphorylated Smad2/3 levels in comparison with wild type cells. Besides, TGF-β treatment incremented the amount of phosphorylated Smad2/3 in β3 integrin competent and deficient cells, albeit at lower extent in β3 integrin competent cells. The presence of TGF-β signaling in β3-integrin deficient cells did not restore the protrusion of degradative 3D structures on collagen DQ (Fig 2D). Therefore from this data we conclude that this pathway is not the responsible of impaired invadopodia formation in β3 integrin deficient cells.

In order to extend these results to other NSCLC cell lines, we used H1299 and A549 NSCLC cells, which express β3 integrin on their cell membrane, and tested them for invadopodia formation by gelatin degrading assays and actin staining by immunofluorescence. As it is shown in S2 Fig, these cells are capable of assembling invadopodia, with proteolytic activity, and these invasive structures are significantly reduced after treatment with β3 integrin blocking mAbs. Therefore, NSCLC cells are capable of producing invadopodia in a β3 integrin-dependent manner.

**Fig 3:**
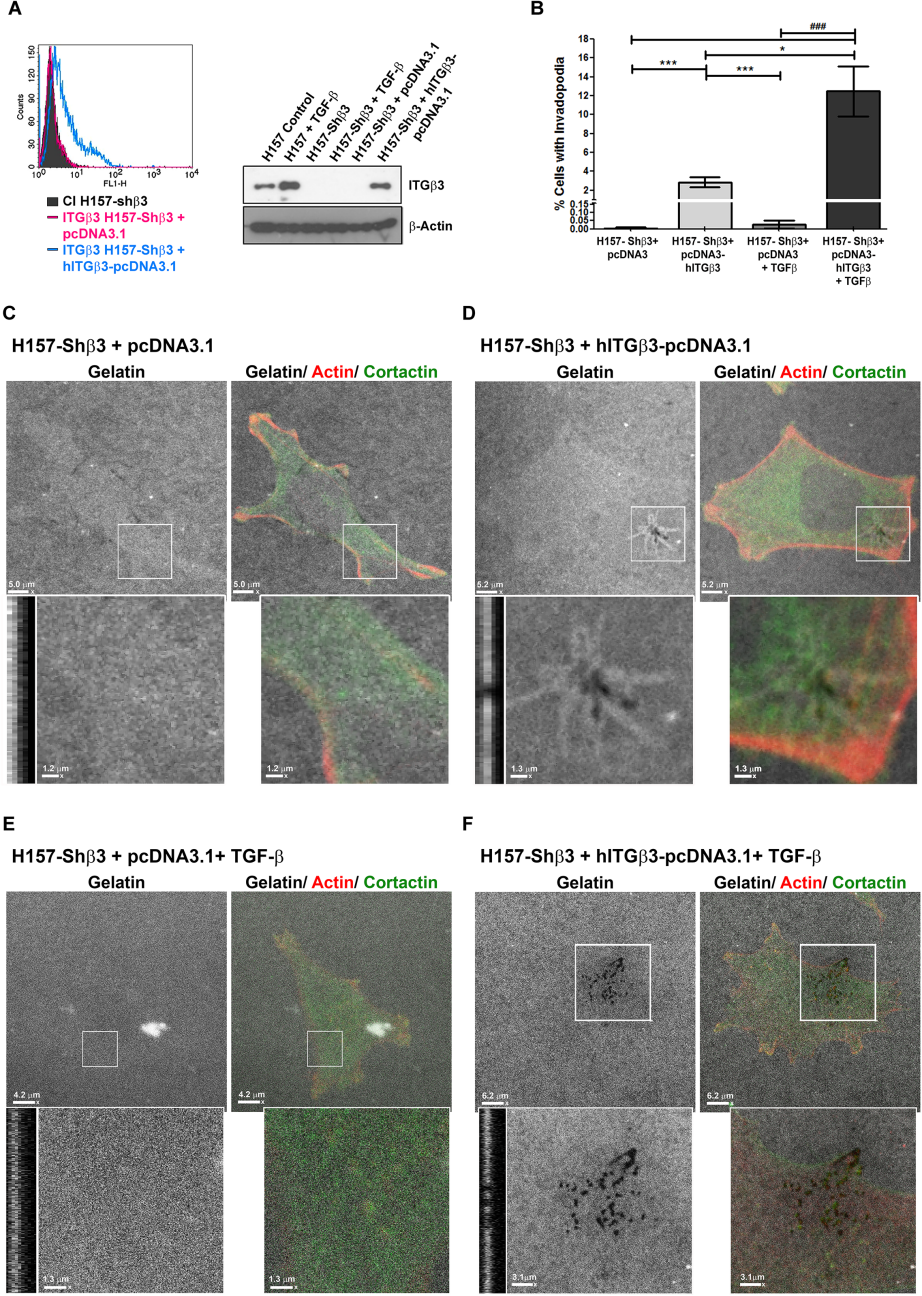
Transfection with β3 integrin-expression vectors restores invadopodia formation in β3 integrin H157 silenced cells. **(A)** Flow cytometry and Western blot analysis of β3 integrin expression after transfection of H157 β3-integrin deficient with pcDNA3 y pcDNA3-hITGβ3 expression vectors c. β-Actin was used in Western blot as loading control. **B)** Quantification of the percentages of cells with gelatin-degrading invadopodia in cells that re-express β3 integrin treated or not with TGF-β. Data represent mean ± SEM of cells obtained from at least three different experiments. Four fields and a total of 25 cells were analysed per experiment. Significant differences were analysed by the Mann-Whitney U test for non-parametric samples. * p< 0.05, *** p< 0.001. *vs*. H157-Shβ3+ pcDNA3-hITGβ3. ### p< 0.001. *vs*. H157-Shβ3+ pcDNA3-hITGβ3+ TGF-β. **(C-F)** Representative images obtained from confocal-laser microscopy of actin (red), cortactin (green), fluorescent gelatin (grey), and degradation areas generated by cells transiently transfected with the expression vector pcDNA3-hITGβ3 **(D, F)** and cells transfected with pcDNA3.1 control vector **(C, E)**. Cells in panels **E** and **F** were treated with TGF-β for 5 days before seeding over gelatin covered plates. Scale bars are 5.2 μm for pcDNA3-hITGβ3 **(D**), 5.0 μm for pcDNA3.1 **(C),** 6.2 μm for pcDNA3-hITGβ3+ TGF-β **(F)** and 4.0 μm for pcDNA3.1+ TGF-β. **(E)**. Amplified areas (white boxes) and z planes are shown below and evidence the degradation areas generated under transfected cells. Scale bars of the amplified areas are 1.3 μm for pcDNA3-hITGβ3 **(D)**, 1.2 μm for pcDNA3.1 **(C)**, 3.1 μm for pcDNA3-hITGβ3+ TGF-β **(F)** and 1.3 μm for pcDNA3.1+ TGF-β **(E)**.

To further prove the importance of β3 integrin expression for mature invadopodia formation, we transfected β3 integrin silenced H157 cells with the β3 integrin expression vector pcDNA3.1-hITGβ3. Integrin re-expression was demonstrated by flow cytometry and Western blotting (Fig 3A) and invadopodia activity was tested with a gelatin degradation assay. As shown in Fig 3B, the gelatin-degrading activity was fully recovered after re-installing β3 integrin expression in silenced cells (Fig 3D) and was not observed in cells transfected with empty vector (Fig 3C). As expected, TGF-β treatment did not induce invadopodia in β3 integrin deficient cells but was able to induce evident invadopodia when the same integrin had been re-expressed in previously silenced cells (Fig 3F and 3G).

### β3 integrin silenced cells overcome deficient cell adhesion but are unable to protrude invadopodia

**Fig 4:**
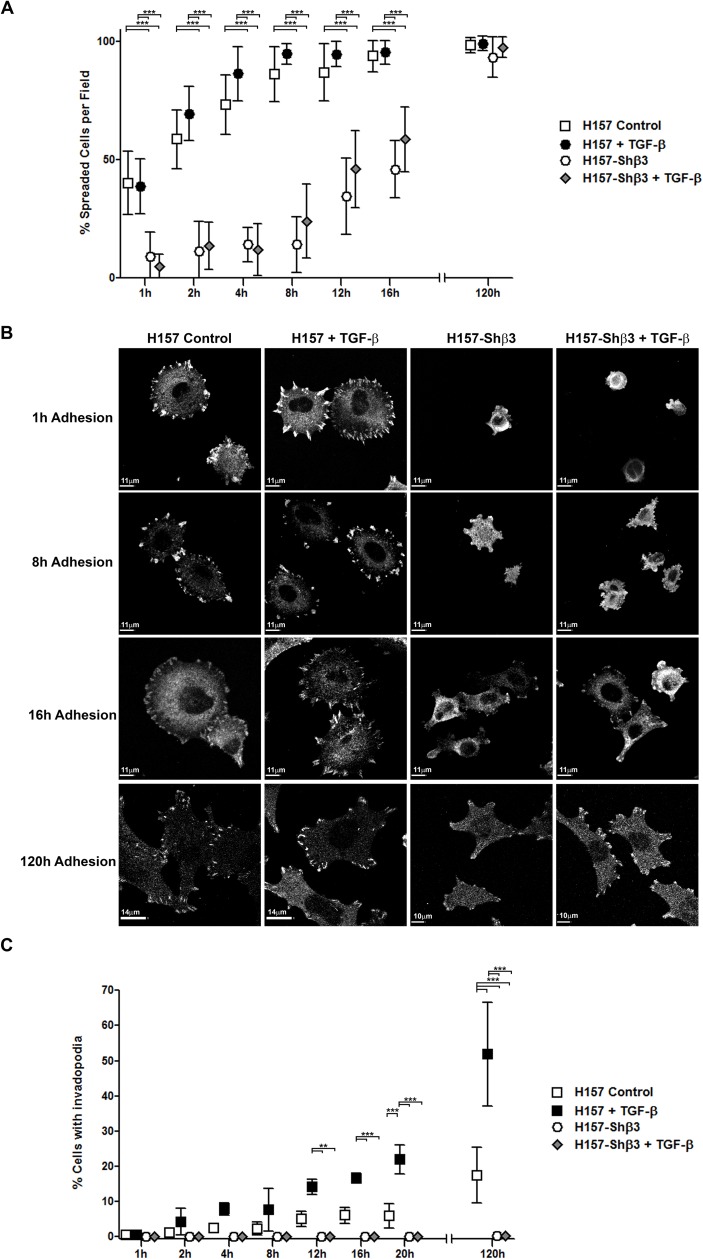
β3 integrin deficient H157-cells can overcome impaired cell adhesion but are unable to form invadopodia. **(A)** Time-course staining of cell adhesion to gelatin coated coverglasses of β3 integrin proficient and deficient H157 cells exposed or not to TGF-β. Adhesion was quantified as percentage of extended cells (height <7μm) from the total number of cells present in a field. Mean ± SEM from three different experiments was calculated and at least 100 cells were analysed per condition. Significant differences were analysed by the Mann-Whitney U test for non-parametric samples. *** p< 0.001. **(B)** Representative microphotographs of cells adhered to gelatin substrates obtained at different time intervals and conditions. Vinculin presence was detected with specific mAb. Scale bars are depicted in each Figure and slightly varied between conditions from 14 to 10 μm. (**C**) Percentage of cells presenting invadopodia at different time points in cells treated as in A. Data represent mean ± SM from three independent experiments in which at least 100 cells were analysed per condition. Significant differences were analysed by the Mann-Whitney U test for non-parametric samples. ** p< 0.01 and *** p< 0.001.

To ascertain whether the inhibition of invadopodia formation in cells defective in β3 integrin expression was a consequence of defective cell adhesion, we used confocal microscopy to capture the formation of vinculin-positive focal adhesion contacts, and quantified the percentage of adhered cells in cultures of cells competent or not for β3 integrin expression seeded over gelatin layers. In parallel experiments, we evaluated the same cells for invadopodia formation. As expected, we observed severely delayed cell adhesion in cells that did not express β3 integrin (Fig 4A); while control cells reached 50% of cell adhesion 1 hour after seeding, β3 integrin deficient cells reached this level of adhesion as late as 16 hours after seeding. Altered cell adhesion was characterized by lower cell spreading and less defined distribution of focal adhesion contacts detected by vinculin staining (Fig 4B). Nonetheless, five days after seeding, both integrin proficient and deficient cells reached 100% adhesion, most probably as a result of the compensatory intervention of other integrins expressed on the cell surface of β3 integrin deficient H157 NSCLC cells. In contrast, we were unable to detect invadopodia in β3 integrin deficient cells at any time point assayed, even five days after initial seeding (Fig 4C). These results demonstrate that while β3 integrin is dispensable for cell adhesion, its presence is required for the onset of invadopodia activity. Therefore, we can postulate that cell adhesion and invadopodia formation are sequential but independent processes mediated by β3 integrin in H157 NSCLC.

### β3 integrin is present in invadopodia core and adhesion ring

**Fig 5:**
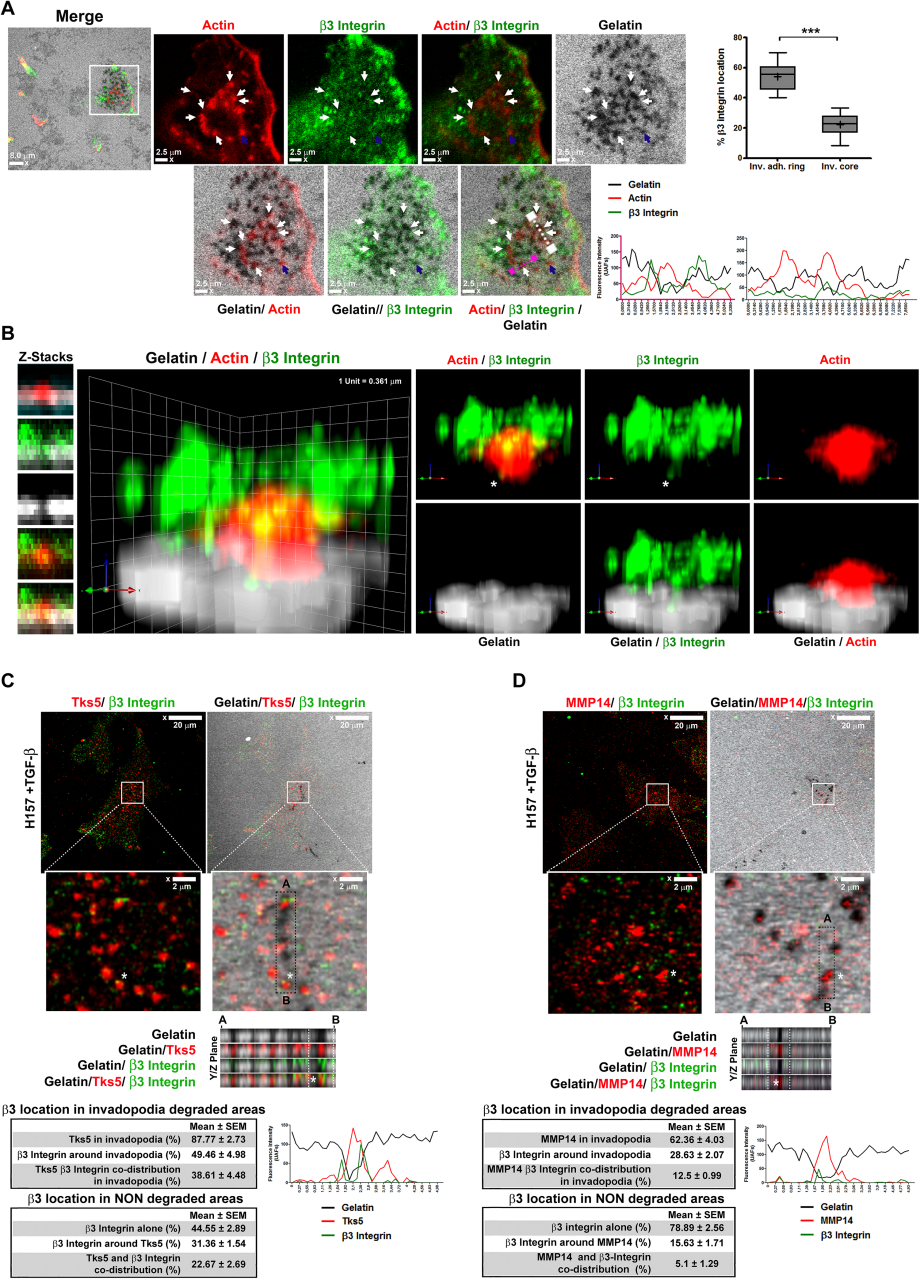
β3 integrin is detected in the invadopodia of NSCLC cells. **(A)** Representative confocal-laser microscopy images of actin (red) and β3 integrin (green) staining in invadopodia of NSCLC H157 cells that had been cultured onto fluorescent gelatin-coated coverglasses (grey). Arrows denote β3 integrin localization in the invadopodia. Scale bars 2.5 μm. Intensity profiles show the fluorescence intensity of actin, β3 integrin, and gelatin in the regions delimited by the white and purple lines depicted on the amplified areas. Box and whiskers plot shows the percentages of β3 integrin located in close contact with the actin-core or with the adhesion ring of invadopodia formed by NSCLC cells in three independent experiments. Significant differences were analysed by the Mann-Whitney U test for non-parametric samples. A minimum of 17 cells were analyzed per condition. +: mean; whiskers: 5th and 95th percentiles. *** p <0.001. **(B)** 3D reconstruction of an amplified image of a β3 integrin positive invadopodium indicated by a blue arrow in 5A. In the image actin is depicted in red, β3 integrin in green, and gelatin in grey. White asterisk indicates the β3 integrin location within gelatin-degraded areas near the center of the invadopodium. Detection of Tks5 (red in **C**) or MMP14 (red in **D**) co-staining with β3 integrin (green) by confocal microscopy performed on cells adhered to gelatin covered substrates. Scale bar 20 μm. Amplified areas (white boxes, scale bar 2 μm) and a XY plane are shown below. Intensity profiles of the region denoted by a white asterisk are shown below the microphotographs. Tables represent the percentages of Tks5 or Mmp14 co-staining with integrin β3 in invadopodia and in non-degraded areas. Mean ± SEM from three different experiments are shown. At least 25 fields were analyzed and approximately 300 invadopodia were analyzed per experiment.

To investigate whether β3 integrin was located in invadopodia, we immuno-stained TGF-β treated H157 cells, which had formed invadopodia on gelatin coated surfaces, with antibodies against β3 integrin and actin. We observed instances of invadopodia that showed co-staining for actin and β3 integrin surrounding the degrading spots (invadopodia adhesion ring) and also in close contact with central actin (invadopodia core) (Fig 5A and 5B). Nevertheless, this integrin was present only in a fraction of total analyzed invadopodia and, therefore, did not seem to be a permanent structural component (Fig 5A). Then, to compare β3 integrin distribution in mature and immature invadopodia, we detected its co-staining with Mmp14 or Tks5, markers of late or early invadopodia respectively. Results shown in Fig 5C, demonstrate co-staining of Tks5 and β3 integrin in 38% of invadopodia located over gelatin degraded areas, while only 12% of invadopodia show positive staining for Mmp14 and β3 integrin (Fig 5D). When we checked for β3 integrin co-staining with Tks5 or Mmp14 in non-degraded areas, we observed a percentage of Tks5 and β3 integrin colocalization of 22%. This was not surprising since Tks5 is one of the adaptor components of Src signal transduction pathways. In contrast, β3 integrin colocalization or association with Mmp14 was reduced to the 5%.

### β3 integrin activates different signaling pathways to induce invadopodia formation

**Fig 6:**
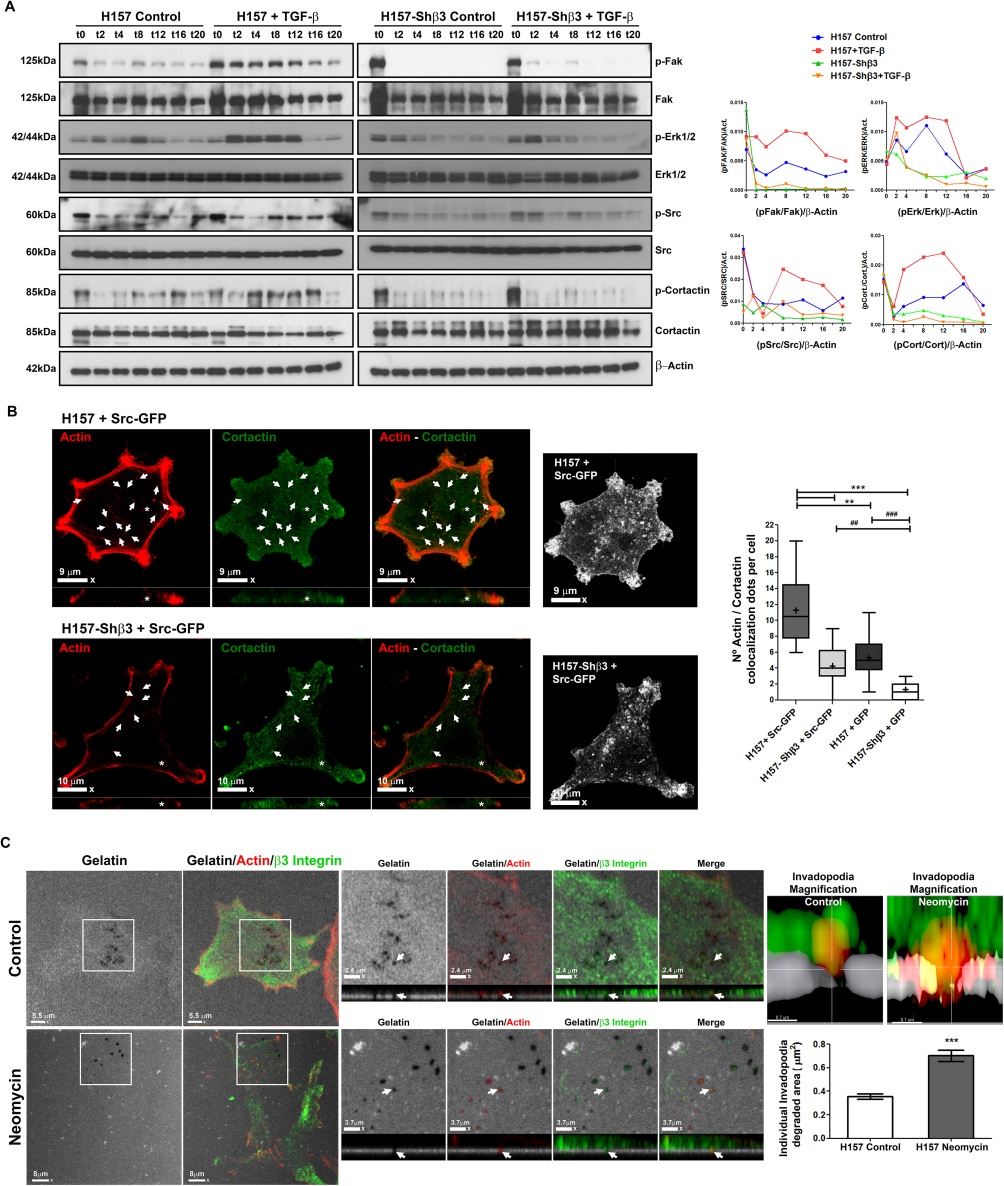
Signal pathways associated to invadopodia formation in H157 NSCLC. **(A)** Detection of Fak, Erk1/2, Src, and cortactin phosphorylation by Western blot (left images) and quantification of Western blot signal intensity (right graphic) in cells competent and deficient in β3 integrin expression treated or not with TGF-β and seeded on gelatin coated plates. **(B)** Detection by confocal microscopy of actin (red) and cortactin (green) distribution in H157 and β3 integrin deficient cells transiently transfected to express SRC-GFP and cultured onto gelatin-coated coverglasses. White arrows and asterisks denote cortactin-actin colocalization dots in ventral actin puncta. Scale bars are 9 μm for H157+SRC-GFP and 10 μm for H157Shβ3+SRC-GFP. The box and whiskers plot represents mean number of actin/cortactin dots per cell. Data was obtained from three different experiments. Significant differences were analysed by one-way ANOVA followed by post-hoc T3 Dunnett tests for multiple comparisons. At least 28 cells were analyzed per condition. +: mean; whiskers: 5th and 95th percentiles. ** p< 0.01, *** p< 0.001. *vs*. H157 + pSRC-GFP. ### p< 0.001. *vs*. H157-Shβ3+ p-GFP. **(C)** Confocal-laser microscopy detection of actin (red), β3 integrin (green) and gelatine (grey) in the invadopodia of NSCLC H157 cells pre-treated, or not, with the PLC inhibitor neomycin (20 mM). Magnifications and Z-stacks of the region boxed in left most microphotograph is represented to its right. White arrows denote β3 integrin localization in the invadopodia. Scale bars 2,4 and 3.7 μm. Invadopodia magnification show fluorescence intensity of actin, β3 integrin, and gelatin of the regions denoted by white arrows. Graphic located at the bottom depicts quantifications of mean degraded area under each individual invadopodia. For statistical analyses Mann-Whitney U test for non-parametric samples was used. At least 50 degraded areas were analysed per sample. *** p<0.001.

Many signaling pathways have been associated to podosome and invadopodia formation in normal and cancer cells, respectively. We have analyzed the activation of adhesion-mediated signaling pathways related to invadopodia formation such as Fak, Src and Erk in cells expressing or not β3 integrin, exposed or not to TGF-β. We also detected the phosphorylation of cortactin, a structural protein from invadopodia. Fig 6A show a representative result in which the time 0 corresponds to protein expression in trypsinized cells in suspension. We obtained high levels of protein phosphorylation at time 0 for all the proteins tested as it has been described previously [29,30,31]. The phosphorylation levels diminished during the first 2-4h and increased at latter time points. As expected, TGF-β treatment incremented the phosphorylation of all the proteins analyzed in β3 integrin competent cells. In contrast, those cells defective in β3 integrin expression showed low amounts of phosphorylated FAK, ERK, Src and cortactin irrespective of TGF-β exposure (Fig 6A). Again, this results support the importance of integrin β3 presence for correct invadopodia formation, in this case, facilitating signal transduction.

Next, we tested whether the inhibition of invadopodia activity observed after β3 integrin silencing could be overcome by over-expression of SRC, an important signal transduction protein that leads to a higher number of invadopodia when it is over-expressed [32]. To this end, we transfected H157 NSCLC cells proficient and deficient in β3 integrin with a SRC-GFP expression vector and a GFP expression vector and analyzed the formation of invadopodia detected as cortactin and actin positive spots in the ventral side of cells, seeded on gelatin layers. As shown in Fig 6B and S2C Fig, β3 integrin deficient cells presented significantly lower colocalization of cortactin on actin puncta than controls despite being transfected with SRC-expression vectors, although they formed some invadopodia most probably by mechanisms independent of β3 integrin expression such as for example integrin β1 profusely described to be present in invadopodia As expected, cells transfected with empty vector also showed lower number of invadopodia in controls than SRC transfected cells. Importantly, invadopodia were absent in cells defective in β3 integrin expression, corroborating our previous results. Therefore, expression of β3 integrin is important, not only for initial Fak-mediated cell adhesion, but also for the formation of invadopodia, even in forced conditions of invadopodia structuration by TGF-β exposure and SRC over-expression.

Finally, we used neomycin, an inhibitor of PIP_2_ signaling as a means to stabilize invadopodia and observe whether β3 integrin deficient cell showed less stable invadopodia. [33]. As expected, when we treated H157 cells with neomycin we are able to detect important increments in invadopodia size and a clear accumulation of β3 integrin on degradation spots adding more evidences for the presence of this integrin in invadopodia (Fig 6C).

## Discussion

Integrin αvβ3 belongs to a family of membrane receptors whose main function is to mediate the connection between the cell surface and the extracellular matrix [34]. Although β3 integrin is expressed at low levels during homeostasis it is up-regulated during tissue remodeling. For example, β3 integrin is highly expressed on the surface of osteoclasts, angiogenic endothelial cells, luminal progenitor cells of the developing mammary gland [35], and smooth muscle cells during vascular remodeling [36]. In addition, the expression of β3 integrin on the cell surface is strongly activated in cells undergoing epithelial-to-mesenchymal transition during tumor progression [37]. Even more, it importantly contributes to tumor stemness and resistance to EGFR inhibition [38]. Thus, β3 integrin actively participates in events that require matrix degradation and cellular migration.

We have previously reported that blockade of β3 integrin expression in NSCLC tumors severely impaired their ability to metastasize towards the lymph nodes [15]. In this study we provide molecular insights on the role played by β3 integrin in NSCLC tissue invasion and demonstrate its presence in ventral invadopodia. The expression of β3 integrin and the αvβ3 heterodimer in tumor cells have been associated with poor prognosis and increased metastasis in several carcinoma types, including osteosarcoma, pancreatic cancer, melanoma, and breast cancers [39,40,41].

In our experimental model, functional or genetic blockade of β3 integrin expression completely impeded invadopodia formation and ECM degrading activity of lung carcinoma cells. Our data suggest that β3 integrin may affect invadopodia formation in two different ways. On the one hand this integrin might contribute to the establishment of initial focal adhesion contacts that signals towards the cytoskeleton. On the other, it could transiently participate in the adhesion rings that contribute to invadopodia maturation. In fact, our results demonstrate that β3 integrin silencing, or blockade, induces a significant delay on the initial cell adhesion to the substrate that is accompanied with lower levels of Fak and Src phosphorylation. Recently we have also demonstrated that integrin blockade induces a decrease in the cell migration capacity and modifications of the migration phonotype of cells in 3D matrices [16]. Focal adhesion structures are sensors of extracellular rigidity, and transduce ECM stiffness towards the cellular contractile apparatus through the activation of Fak and Src pathways, among others. Assembly and disassembly of membrane located focal adhesions is key for the correct arrangement of ventral invasive structures [42]. Even more, the elements that constitute both adhesion structures seem to compete with each other [43]. Thus, the decreased phosphorylation of Fak and Src observed in our experiments along with the non-permanent presence of β3 integrin in invadopodia may be another example of this phenomenon. Although severely delayed, H157 β3 integrin-deficient cells finally adhered to the substrate, most probably due to the compensatory action of other integrins expressed by these cells [44]. In contrast, we were unable to detect invadopodia at any time in β3 integrin-deficient cells. Therefore, the participation of β3 integrin in invadopodia formation in our system is not restricted to facilitate initial cell adhesion. In fact, we provide confocal image-based evidences of β3 integrin presence in invadopodia formed by NSCLC.

The presence of β3 integrin in ECM degrading structures formed by non-transformed cells, named podosomes, had already been described [18]. In these cells, β3 integrin mediates the initial collection of podosomes to the bone-facing plasma membrane but it disappears from this location in the mature ECM-resorbing osteoclast [45]. In our experiment we observed β3 integrin colocalization with Mmp14 and Tks5 in invadopodia. Integrin associated mainly to the early invadopodia marker Tks5, but it also associates to the mature marker Mmp14 albeit at lower percentage. This is, to our knowledge, the first evidence of the participation of β3 integrin in the structuration and activity of invadopodia in transformed cells.

Our data is endorsed by the results obtained by Ponceau and coworkers in podosomes of HMEC-1 cells, where they observed transitory localization of β3 integrin and suggested its importance in the formation of the structure [46]. They also observed that β3 integrin location in podosomes can be stabilized by αII-spectrin. In our conditions the location of β3 integrin in invadopodia would be stabilized by neomycin and by subsequent increments in PIP_2._ The inhibition of PLC with neomycin increases PIP_2_ in the cell, blocking its degradation to diacylglycelol (DAG) and inositol 1,4,5-triphosphate (IP_3_) [47]. Neomycin also increases pErk1/2 levels in the cells [48], which is a signal pathway associated to invadopodia formation and TGF-β signaling [27].

We have also observed a double localization for β3 integrin in the invadopodium structure. Different roles have been ascribed to β3 integrin, from cell attachment to the degradation of the extracellular matrix [49]. Its presence in the adhesion ring could be associated to a mechano-transductional role in these structures [12,50], while in the invadopodia core location β3 integrin would contribute to the maturation of pro-Mmp-2 by Mmp14 -mediated cleavage [51,52]. We have also observed that Mmp2 is the main gelatinase secreted by H157 lung cancer cell line after TGF-β treatment in NSCLC.

**Fig 7:**
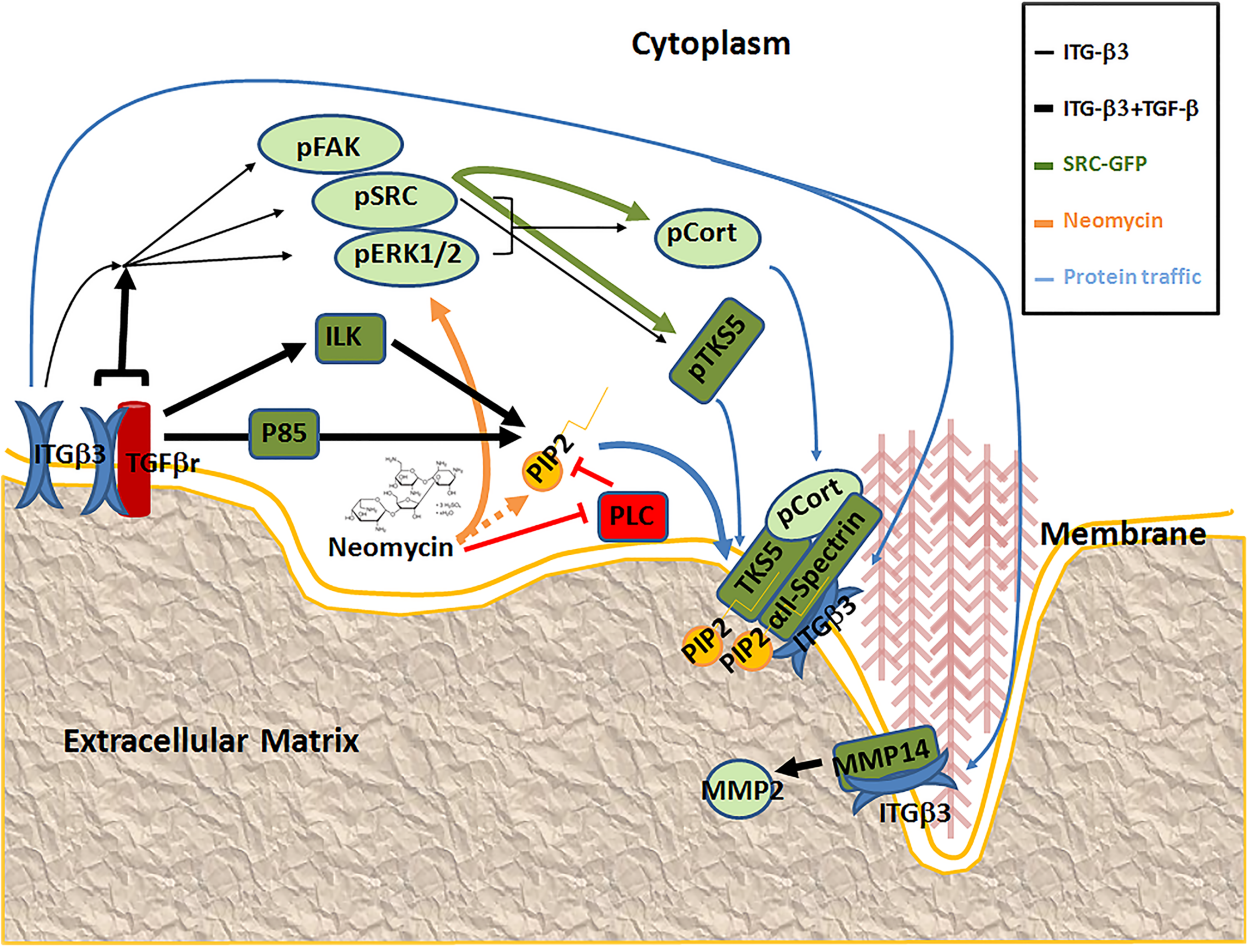
Schematic representation of signal pathways associated to invadopodia formation in H157 NSCLC. We have detected that β3 integrin activates signal pathways in H157 NSCLC cells (thin black arrows) such as Fak, Src, Erk, and in consequence, Cortactin (light green ovals) to form invadopodia structures. It could also activate other proteins required for invadopodia (dark green boxes). H157 cells exposed to TGF-β increase integrin clustering, and its association with TGF-β receptors, to accelerate the activation of signal pathways (wide black arrows) associated to invadopodia formation. In these conditions the cells would present higher levels of PIP_2_ to recruit Tks5, Spectrin and β3 integrin to form the invadopodia in the cell membrane. Src is a protein whose expression had been associated to invadosome formation, so its over-expression would activate signals to induce invadopodia formation (wide green arrows). Red lines represent an inhibitory effect over the indicated protein. Neomycin inhibits PLC, which reduces the levels of PIP_2_, so Neomycin increases PIP_2_ levels indirectly (discontinuous orange arrow). Neomycin also activates Erk1-2 signalling (continuous orange arrow). Blue arrows show the traffic of the proteins to their functional location in invadopodia.

Invadopodia formation is a process dependent on multiple signal pathways. Cortactin, and its phosphorylated form [53], are critical factors associated with invadopodia formation [54]. The C-terminus domain of cortactin presents the main susceptible sites of phosphorylation by Src family, Erk1 / 2, and other kinases [53]. Src also phosphorylates other important proteins associated with Tks5 [55,56]. In agreement with the literature, our results show an increased level of pCortactin, pSrc, and pErk1 / 2 in TGF-β treated cells, while they were not detected in β3 integrin-silenced cells. High levels of pErk1/2 associated to TGF-β treatment have been already demonstrated in tumor cells [27]. Tks5 is a protein whose interaction with PIP_2_ allows its anchoring to the cell membrane, cortactin and actin machinery recruitment and, finally, invadopodia formation [57]. PIP_2_ will also recruit αII-spectrin, which would recruit β3 integrin to the invadopodia. This hypothesis is also consistent with the fact that spectrin interacts with PtdIns(4,5)P_2_ through its PH domain [58]. In cancer cells, TGF-β signaling can activate the PI3K-Akt pathway either directly or indirectly, to increase PIP_2_ and PIP_3_ to recruit proteins with lipid-binding domains to the plasma membrane [59]. Our proposed model of invadopodia formation can be reviewed in Fig 7. It shows the signaling pathways and the methodological approaches to understand the formation of invadopodia in H157 NSCLC, and the role of β3 integrin in the process.

In addition, β3 integrin can act as co-receptor for several growth factors such as EGF, FGF, and TGF-β [60,61,62] through binding to their ligands in order to facilitate their activation. Even more, TGF-β-mediated induction of β3 integrin has been described as part of a positive feed-back loop, in which β3 integrin increments TGF-β activation by binding to the RGD domains in a complex formed by TGF-β and the latent associated peptide (LAP). This activation contributes to TGF-β-stimulated cancer metastasis [28]. In summary, all these activities can be at work during invadopodia-mediated ECM degradation in NSCLC.

Although other studies have not found a relevant role for β3 integrin in invadopodia regulation [12,18,23,63], our results are in concordance with evidences published on integrin location in adhesion rings of invadosomes [12,46], and on the functional contribution of β3 integrin to this structure [22], showing that β3 integrin blockade, or gene silencing, completely prevents invadopodia formation in NSCLC. This discrepancy can be a consequence of the different integrin blockade strategies used, alternative integrin family members presents in each cell line, or it can emanate from the growth-factors used to induce invadopodia: EGF, HGF, serum, or TGF-β [11,15,22,23]. Furthermore, we show, for the first time, a reduction in the ability to form invadopodia in non TGF-β treated cells, by β3 integrin elimination. These results demonstrate that the role of β3 integrin is much more important for invadopodia formation than just its mechano-transductional role associated to the signaling from growth factors, because the silenced cells still keep TGF-β signaling ability but they are not able to form invadopodia structures. The dialogue between growth factor and adhesive signaling that occurs in carcinoma cells increases aggressiveness and has been extensively exploited as a target for therapy to block metastasis [64]. In this regard, it has been published that radiotherapy increases αvβ3 integrin expression as a survival mechanism in NSCLC H157 and H460 cell lines and, consequently, tumor growth is reduced by a combination of radiotherapy and treatment with the β3 integrin antagonist Cilengitide [65].

Here, we demonstrated that lung carcinoma cells form invadopodia in response to TGF-β exposure. We observed that β3 integrin deficient cells are not able to degrade gelatin-coated surfaces, even though they adhere to the substrate, and activate the classical signaling pathways associated to invadopodia formation. In summary, our results suggest a transitory, but vital, location of this integrin in lung carcinoma cell invadopodia. We also provide evidence for the role of β3 integrin in the onset of invasive structures by lung squamous carcinoma cells providing additional support to the use of β3 integrin blocking agents to combat cancer progression.

## Supporting information

S1 Fig Invadopodia formation and Mmp involvement in H157 NSCLC. **(A)** Representative confocal-laser microscopy images of H157 NSCLC cells seeded onto green fluorescent-labelled gelatin for 20 hours, after being exposed or not for 5 days to TGF-β. Actin was visualized by TRICT-phalloidin staining (red). Scale bars 13 μm. **(B)** Histogram plot quantification of the percentages of cells associated with matrix degradation areas in cells pre-treated with the TGF-βRI inhibitor SB431542. Data represents the quantification of at least three different experiments analysing at least three fields per experiment. Significant differences were analysed by the Student’s t-test for comparison of the mean parametric data. ***p<0.001. **(C)** Representative microphotographs of gelatin-degradation areas in TGF-β activated H157 cells treated or not with the Mmps inhibitor GM6001. Scale bars represent 6.2 and 4.8 μm respectively. Histograms on the right show the fluorescence intensity of actin, cortactin, and gelatin. **(D)** Histogram plots represent the percentage of cells associated with areas of gelatin degradation. At least three different experiments were performed and three fields were analysed per experiment. Significant differences were analyzed by the Mann-Whitney U test for comparison of non-parametric data. * p< 0.01 and ** p< 0.001. **(E)**. Western blot detection of Mmp2 and Mmp9 expression in the supernatant of H157 cells.
(TIF)

S2 Fig β3 integrin blockade affects invadopodia formation in different NSCLC. **(A)** Quantification of cells presenting active degradation areas as a result of β3 integrin blockade in TGF-β treated and untreated H1299 cells. Cells were pre-treated with 13μg of β3 integrin blocking antibody 2 hours before seeding onto gelatin-coated coverglasses. An isotype non-specific IgG treatment was included as the control. Data represent the mean ± SEM of four different experiments analysing at least three fields per experiment. At least 15 fields were analyzed from each condition (n = approximately 130 cells). ** p< 0.01 and *** p< 0.001. Microphotographs in upper panels show representative image from each experimental condition. Scale bars 23 μm. Red asterisks reveal degradation sites on the gelatin matrix. **(B)** Quantification of cells presenting active degradation areas as result of β3 integrin blockade in TGF-β treated and untreated A549 cells. Cells were pre-treated with 1 μg of β3 integrin blocking antibody 2 hours before seeding onto gelatin-coated coverglasses. An isotype non-specific IgG treatment was included as the control. Data represent the mean ± SEM of four different experiments analysing at least three fields per experiment. At least 15 fields were analyzed from each condition (n = approximately 100 cells). * p< 0.01 and ** p< 0.001. Microphotographs in upper panels show representative image from each experimental condition. Scale bars 23 μm. Red asterisks reveal degradation sites on the gelatin matrix. **(C)** Detection by confocal microscopy of actin (red), cortactin (green) co-staining and Src (grey) distribution in H157 and β3 integrin deficient cells transiently transfected to express -GFP and cultured onto gelatin-coated coverglasses. White arrowheads and asterisk denote cortactin-actin colocalization with ventral actin puncta. Scale bars are 5,8 μm for H157+ GFP and 6,2 μm for H157Shβ3+ GFP.
(TIF)
