# Supplementary figures and images for: β3 integrin expression is required for invadopodia-mediated ECM degradation in lung carcinoma cells
Source: PLoS One. 2017 Aug 2;12(8):e0181579. doi: 10.1371/journal.pone.0181579 (PMC5540285; doi:10.1371/journal.pone.0181579)

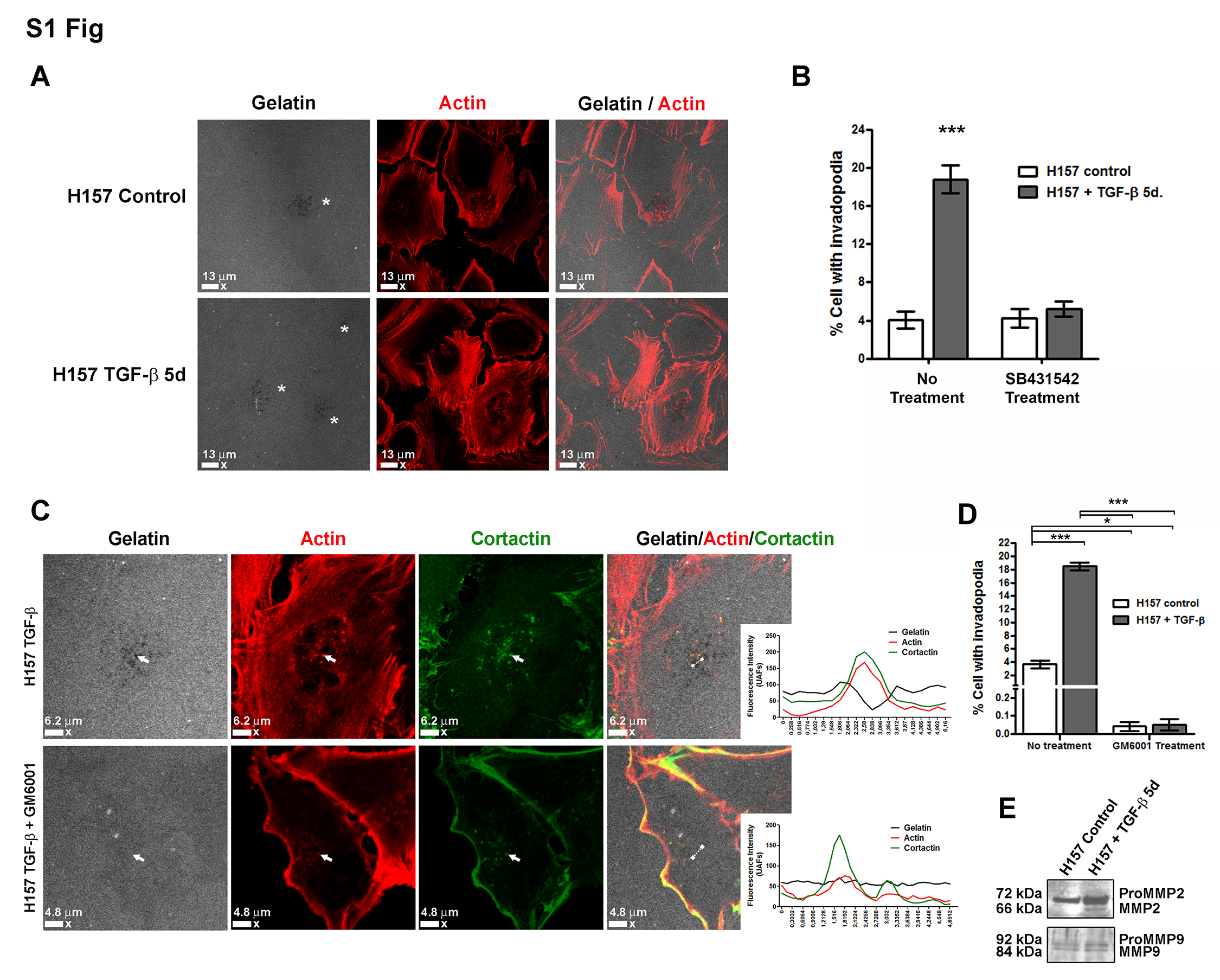

Supplement: S1 Fig — (A) Representative confocal-laser microscopy images of H157 NSCLC cells seeded onto green fluorescent-labelled gelatin for 20 hours, after being exposed or not for 5 days to TGF-β. Actin was visualized by TRICT-phalloidin staining (red). Scale bars 13 μm. (B) Histogram plot quantification of the percentages of cells associated with matrix degradation areas in cells pre-treated with the TGF-βRI inhibitor SB431542. Data represents the quantification of at least three different experiments analysing at least three fields per experiment. Significant differences were analysed by the Student’s t-test for comparison of the mean parametric data. ***p<0.001. (C) Representative microphotographs of gelatin-degradation areas in TGF-β activated H157 cells treated or not with the Mmps inhibitor GM6001. Scale bars represent 6.2 and 4.8 μm respectively. Histograms on the right show the fluorescence intensity of actin, cortactin, and gelatin. (D) Histogram plots represent the percentage of cells associated with areas of gelatin degradation. At least three different experiments were performed and three fields were analysed per experiment. Significant differences were analyzed by the Mann-Whitney U test for comparison of non-parametric data. * p< 0.01 and ** p< 0.001. (E). Western blot detection of Mmp2 and Mmp9 expression in the supernatant of H157 cells. (TIF) [file pone.0181579.s001.tif]

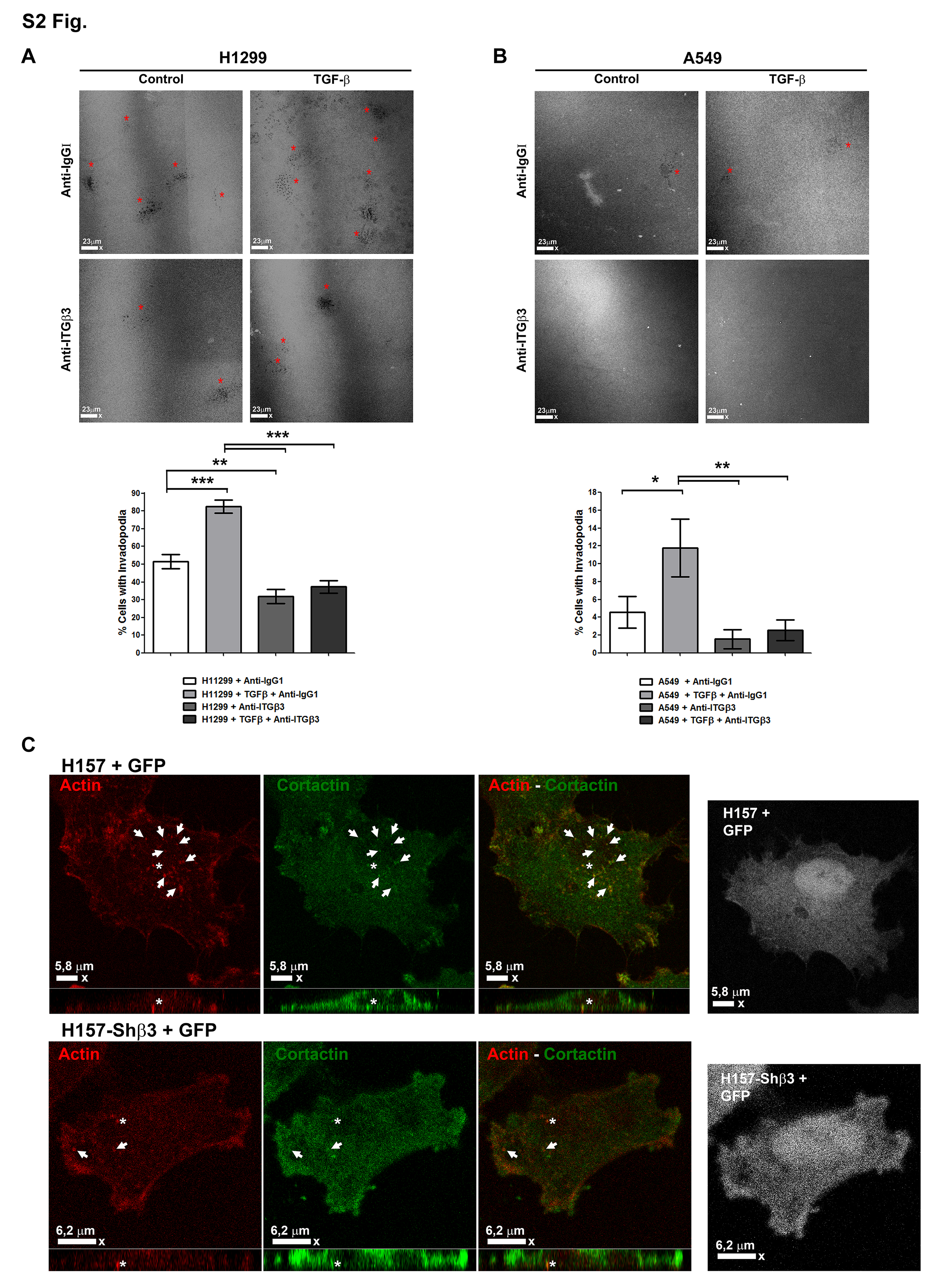

Supplement: S2 Fig — (A) Quantification of cells presenting active degradation areas as a result of β3 integrin blockade in TGF-β treated and untreated H1299 cells. Cells were pre-treated with 13μg of β3 integrin blocking antibody 2 hours before seeding onto gelatin-coated coverglasses. An isotype non-specific IgG treatment was included as the control. Data represent the mean ± SEM of four different experiments analysing at least three fields per experiment. At least 15 fields were analyzed from each condition (n = approximately 130 cells). ** p< 0.01 and *** p< 0.001. Microphotographs in upper panels show representative image from each experimental condition. Scale bars 23 μm. Red asterisks reveal degradation sites on the gelatin matrix. (B) Quantification of cells presenting active degradation areas as result of β3 integrin blockade in TGF-β treated and untreated A549 cells. Cells were pre-treated with 1 μg of β3 integrin blocking antibody 2 hours before seeding onto gelatin-coated coverglasses. An isotype non-specific IgG treatment was included as the control. Data represent the mean ± SEM of four different experiments analysing at least three fields per experiment. At least 15 fields were analyzed from each condition (n = approximately 100 cells). * p< 0.01 and ** p< 0.001. Microphotographs in upper panels show representative image from each experimental condition. Scale bars 23 μm. Red asterisks reveal degradation sites on the gelatin matrix. (C) Detection by confocal microscopy of actin (red), cortactin (green) co-staining and Src (grey) distribution in H157 and β3 integrin deficient cells transiently transfected to express -GFP and cultured onto gelatin-coated coverglasses. White arrowheads and asterisk denote cortactin-actin colocalization with ventral actin puncta. Scale bars are 5,8 μm for H157+ GFP and 6,2 μm for H157Shβ3+ GFP. (TIF) [file pone.0181579.s002.tif]
